# Adherence to Hypertension Medications and Lifestyle Recommendations among Underserved African American Middle-Aged and Older Adults

**DOI:** 10.3390/ijerph17186538

**Published:** 2020-09-08

**Authors:** Edward Adinkrah, Mohsen Bazargan, Cheryl Wisseh, Shervin Assari

**Affiliations:** 1 Department of Family Medicine, Charles R. Drew University of Medicine and Science, Los Angeles, CA 90059, USA; edwardadinkrah@cdrewu.edu (E.A.); Mohsenbazargan@cdrewu.edu (M.B.); cWisseh@westcoastuniversity.edu (C.W.); 2 Department of Family Medicine, University of California Los Angeles (UCLA), Los Angeles, CA 90059, USA; 3 Department of Pharmacy Practice, West Coast University School of Pharmacy, Los Angeles, CA 90004, USA

**Keywords:** race, hypertension, adherence, lifestyle

## Abstract

*Background*: For African American middle-aged and older adults with hypertension, poor adherence to medication and lifestyle recommendations is a source of disparity in hypertension outcomes including higher rates of stroke in this population relative to whites. *Aims*: To study demographic, social, behavioral, cognitive, and medical predictors of adherence to medication and lifestyle recommendations among underserved African American middle-aged and older adults with hypertension. *Methods*: This was a community-based cross-sectional survey in South Los Angeles with 338 African American middle-aged and older adults with hypertension who were 55 years or older. Age, gender, continuity of care, comorbidity, financial difficulty, self-rated health, depression, educational attainment, adherence knowledge, and adherence worries were the independent variables. Data was analyzed using linear regression with two outcomes, namely, adherence to medication (measured by the first 9 items of the Blood Pressure Self-Care Scale) and adherence to lifestyle recommendations (measured by the second 9 items of the Blood Pressure Self-Care Scale). *Results*: There were about twice more females than males, with a total mean age of 70 years (range 55–90 years). Various demographic, social, behavioral, and medical factors predicted adherence to medication but not adherence to lifestyle recommendations. Females with hypertension with higher continuity of care, less financial strain, higher knowledge, less negative general beliefs, and concerns about antihypertensive medications had higher adherence to antihypertensive medications. The presence of depressive symptoms, reduced knowledge, and disease management worries were associated with a reduced adherence to lifestyle recommendations. *Conclusions*: There seem to be fewer demographic, social, behavioral, cognitive, and medical factors that explain adherence to lifestyle recommendations than adherence to medication in economically disadvantaged underserved African American middle-aged and older adults with hypertension. More research is needed on factors that impact adherence to lifestyle recommendations of African American middle-aged and older adults with hypertension.

## 1. Introduction

### 1.1. Background

Racial disparities in the prevalence and complications of hypertension among minority older adults, particularly African Americans, have been well documented [1,2,3,4]. There is a worsening trend in the prevalence of cardiovascular diseases, especially hypertension, in the African American older adult population [5]. Recent data demonstrates that more than two-thirds of older African American adult Medicare beneficiaries have hypertension [6]. As a result, adequate hypertension control and disease management among underserved African American minority populations have become a major healthcare priority for the United States [4].

Hypertension is a major risk factor for stroke, one of the leading causes of death in the United States [7]. Although the risk and mortality rates of stroke have declined since the 1960s, racial differences, especially in older African Americans, persist [5]. According to the American Heart Association, African Americans are 80% more likely than Whites to develop a fatal stroke because of poorly controlled hypertension [8]. Bailey and colleagues have highlighted the role of a consistent antihypertensive drug regimen in reducing stroke risk by 11% [9]. Not only nonadherence but also low adherence to antihypertensive medications (AM) increase the risk of stroke [10]. Also, this situation may worsen if not addressed after a stroke event. Poststroke morbidity and mortality are known to worsen with low/poor antihypertensive medication adherence [11].

Nonadherence to two aspects of hypertension management, namely prescribed drug regimens and lifestyle recommendations, are the two major challenges that lead to uncontrolled, resistant, and complicated hypertension (heart failure, stroke, and renal failure) [12,13,14,15,16,17,18,19,20,21,22,23]. There is strong evidence that shows poor adherence to antihypertensive medications is associated with uncontrolled blood pressure among African Americans [24]. Poor adherence to cardiovascular medications, antihypertensive agents, and statins among older African American adults is also well documented [4,5,6,7,8,9,10,11,12,13,14,15,16,17,18,19,20,21,22,23,24,25,26,27,28,29]. For African Americans, poor adherance to antihypertensive medications happens in a context of several other health and medication-related problems. Up to 70% of older African Americans use inappropriate medications [30], and 30% use at least ten medications to treat chronic conditions [31].

Self-management, which includes medication adherence, also encompasses lifestyle modification behaviors such as healthy dietary habits, exercise and weight loss, limited or no alcohol consumption, and avoidance of the use of tobacco products [32]. Numerous studies have documented that effective self-care behavior or self-management can substantially reduce hypertension complications [33]. Moreover, lifestyle modifications/recommendations are known to considerably decrease blood pressure, sometimes without the need for blood-pressure-lowering medication [33].

One of the main purposes of self-management is to enable patients with hypertension to be closely engaged in self-care which is an essential requirement for long-term blood pressure control [34]. However, the rate of self-management is still considerably low among African American older adults with hypertension [35] as African Americans have been documented to have low adherence levels to lifestyle recommendations [36,37] and to engage in unhealthy behaviors such as physical inactivity, consumption of high fat and sodium containing foods, and tobacco and alcohol use [38,39]. This supports studies by psychological scientists who have indicated that individuals increasingly struggle with negative behaviors and find it hard to adopt and maintain healthier behaviors [40].

Thus, we hypothesized direct and positive associations between self-care management and adherence to lifestyle recommendations and medications in hypertensive African American middle aged and older adults living in South Central Los Angeles [41].

### 1.2. Aim

This study seeks to identify demographic, social, behavioral, cognitive, and medical correlates of adherence to medication and lifestyle recommendations among medically underserved African American middle-aged and older adults with hypertension in South Central Los Angeles.

## 2. Materials and Methods

### 2.1. Design and Setting

This study employed a cross-sectional study design. Non-probabilistic convenience sampling was used to recruit African American older adults from low income areas in Service Planning Area 6 (SPA 6) of Los Angeles County. Considered among the most underserved regions in the nation, SPA 6 includes low-income communities of South-Central Los Angeles, like Athens, Crenshaw, Lynwood, and Paramount. More than one million Americans live in SPA 6 and are disproportionately affected by health disparities [42]. This study was performed between 2015 and 2018.

### 2.2. Human Subjects Protection

This study was assessed and ratified by the Institutional Review Board of Charles R. Drew University of Medicine and Science (IRB 14-12-2450-05). A statement of the purpose and nature of the study was provided to all prospective participants, and they were assured that participation was voluntary. Subjects were asked to sign informed consent before participating in the study. The consent form emphasized that participants’ responses to all questions would be kept confidential. Participants were also given the option to refuse to answer any or all questions that they felt were too personal.

### 2.3. Sampling

Respondents were included if they indicated that they have ever been diagnosed with hypertension (338 participants), were African American, were 55 years of age or more, were able to finish an interview in English, and lived in SPA 6. Individuals who were institutionalized, enrolled in a clinical trial, or had declining cognition were excluded. This sampling included 338 African American adults aged 55 years and older.

### 2.4. Measurement

#### 2.4.1. Sociodemographic Factors

Data on sociodemographic factors included age (interval variable), gender (dichotomous variable: female—1 and male—0), and educational attainment (interval variable representing years in school; higher scores suggest higher educational achievement). Financial strain/difficulty was quantified using a 5-point Likert scale (1 = always to 5 = never). Respondents were asked how often in the preceding twelve months they were incapable of (1) “buying the amount of food their family should have”, (2) “buying the clothes they feel their family should have”, (3) “paying their rent/mortgage; (4) paying their monthly bills”, and (5) “making ends meet”. A greater tally was suggestive of more financial strain within the previous twelve-month period (α = 0.934).

#### 2.4.2. Living Arrangement

Participants’ prevailing residential arrangement was measured as a dichotomous variable (living alone—1 and living with someone else—0).

#### 2.4.3. Continuity of Medical Care

Continuity of care was measured using three questions. Respondents were requested to report (1) “what type of place they usually visit to receive medical care (a private doctor’s office/private medical group vs. other settings)”, (2) “whether they usually go to the same place for medical care”, and (3) “whether they are usually seen by the same health provider when they receive medical care”. Responses to question (1) were coded “private office” = 1 and “any other place” = 0. Responses to questions (2) and (3) were coded “yes” = 1 and “no” = 0. The overall tally varied from 0–3, and a higher count suggested greater continuity of care.

#### 2.4.4. Beliefs about Medicines Questionnaire (BMQ)

The 18-item Beliefs about Medicines Questionnaire (BMQ) questionnaire was employed to evaluate beliefs regarding antihypertensive medications [43]. Participants specified the level to which they agreed with each statement on a 5-point Likert scale (strongly disagree = 1; strongly agree = 5). For example, one of the items read, “Doctors use too much antihypertensive medications.” Principal component analysis was used to identify potential factors underlying the 18-item instrument that measures beliefs about medicines. Consistent with previous studies, the varimax rotation produced three distinct factors, explaining 52% of the variance. The initial factor accounted for 22% of the variation, while the second and third factors explained 16% and 14% of the variation. All items had primary loadings over 0.5, and no more than a single item had a cross-loading exceeding 0.3 (“High Blood Pressure—BP medications are a mystery to me”); this item had an acceptable primary loading of 0.50. The first factor was associated with the 8 items that measure general beliefs about medications. Cronbach’s alpha coefficient for these 8 items was calculated to be 0.82. Furthermore, if items were removed, the alpha scores varied from 0.784 to 0.813, signifying that no one item significantly diminished reliability. Therefore, we were convinced that this factor is the best representative index to measure general belief about antihypertensive medications. Again, consistent with previous studies, the second factor produced by the varimax rotation was associated with five items that measures the specific necessity beliefs. The specific necessity beliefs subscale assesses the beliefs of individuals about the necessity of their medications. It is expected that having a strong necessity belief translates into higher medication adherence [44,45]. Finally, the third factor clearly measures the specific concerns about antihypertensive medications. Inversely, strong specific concerns about medications are expected to translate to lower adherence or nonadherence [44,45].

#### 2.4.5. Blood Pressure Knowledge

Blood pressure knowledge was assessed using an instrument that has been validated among African American adults [46]. This instrument asked about specific behaviors such as stress reduction and consumption of low-fat and salt diets that relate to nutrition, alcohol and tobacco use, weight management, physical activity, stress control, and visiting health care providers to control blood pressure. All items on the scale utilize a 5-point Likert scale. Participants indicate their level of agreement with each statement on a 5-point Likert scale, ranging from 1 = strongly agree to 5 = strongly disagree. The average of the summated scale of hypertension knowledge range from 1 to 5, with greater scores reflecting higher levels of knowledge. The Cronbach’s alpha for internal consistency reliability for these items was 0.77.

#### 2.4.6. Self-Rated Health Status

A standard item was used to measure self-perceived health status. Participants were asked the following specific question: “In general, would you say your health is (1) excellent, (2) very good, (3) good, (4) fair, or (5) poor?” This unique item is often used in health research, national surveys, and longitudinal cohorts. Additionally, participants were asked to report whether they had been diagnosed with any of the following major chronic conditions: hypertension, diabetes, stroke, or a heart condition. Finally, participants were asked to report how many different providers they had visited within the last 12 months.

#### 2.4.7. Depressive Symptoms

We utilized the Geriatric Depression Scale (short form) (GDS-SF) to determine the severity and rate of depressive symptoms. This assessment has fifteen items requiring “yes” or “no” responses [43]. The scale offers an overall score which ranges between 0 and 15, where a greater tally suggests more depressive symptomatology. The Geriatric Depression Scale (short form) has exceptional reliability and validity and is broadly applied to assess depressive symptoms in older adults in the society in addition to acute and long-term care settings [45].

#### 2.4.8. Number of Chronic Medical Conditions (CMCs)

We asked respondents whether they had ever been told by a healthcare provider that they have any of the following eleven chronic medical conditions: heart conditions, cancer, asthma, thyroid disorder, chronic obstructive pulmonary disease, diabetes, rheumatoid arthritis, migraine headache, chronic back pain, difficulty sleeping or insomnia, and gastrointestinal-related conditions. While research establishes self-reports as a valid source of chronic medical conditions data, a degree of measurement bias is acknowledged [47].

### 2.5. Dependent Variables

#### Blood Pressure Self-Care Scale

Eighteen items were used to evaluate the blood pressure self-care behaviors. The first 9 items measured adherence to recommended lifestyle modifications for hypertensive adults, and the second 9 items measured adherence to drug regimens. These two instruments have also been developed and validated for use among hypertensive African American adults [46]. Examples of these items include, “I am physically active at least 30 min each day” and “How often do you miss taking your blood pressure medication when you feel unwell?”. All items on the scale utilize a 5-point Likert scale. The average of the summated scale of adherence to medication regimens and lifestyle modifications vary from 1 to 5, with higher scores indicating a higher level of adherence to blood pressure self-care. The Cronbach’s alpha for internal consistency reliability for the behavioral modification scale was 0.71 and for the adherence to medications was 0.84.

### 2.6. Data Analysis

The survey questionnaire has been loaded onto REDCap software (version 9.1.0, Vanderbilt University: Nashville, TN, USA) for “real time” data collection and entry by trained research associates. At the bivariate level, the statistical method used in this study was Pearson’s correlation test. We also used multiple regression techniques to examine the association of independent variables on hypertension self-care indices. Additionally, multiple linear regressions were utilized to assess the relationship between independent variables and self-care indices. Pearson’s correlation test was performed to detect potential harmful multicollinearity among independent variables. We used the SPSS 23.0 (IBM, Armonk, NY, USA) for data analysis with statistical significance at the 0.05 level.

## 3. Results

### 3.1. Descriptive Statistics

Table 1 details the characteristics of the study sample. This study comprised 338 African American older adults between 55 and 96 years of age (69.56 ± 9.25). Among respondents, 65% were 65 years of age or older, 37% were men, and over 24% did not have a high school diploma. Almost 72% of the respondents lived alone.

### 3.2. Health Status and Comorbidity

Regarding health status, only 5% of the sample reported their present health as excellent and more than 43% described their health as fair (33%) or poor (10%). Thirty-three percent of participants had diabetes mellitus, 15% had a stroke, and 28% had heart-related conditions. One out of five suffered from asthma, and more than 57% indicated they currently had back pain. Almost 57% had been treated at least once in an emergency room within the last 12 months, and 27% reported staying overnight at the hospital as a patient, at least once, within last 12 months.

### 3.3. Beliefs and Knowledge about Hypertension

More than 35% of participants indicated that having to take high blood pressure medication worries them. However, 49% indicated that their life would be impossible without high blood pressure medications. More than 30% of participants indicated that they are uncertain (8%) or disagree (23%) that avoiding alcoholic beverages helps to keep their blood pressure within normal limits. Additionally, 16% admitted that they are uncertain or disagree (10%) that avoiding tobacco products helps hypertensive individuals keep their blood pressure within normal limits.

### 3.4. Blood Pressure Self-Care

Only one out of two participants indicated that they always (23%) or most of the time (27%) maintain a low-salt diet. Only 8% indicated that they maintain a low-fat diet. Almost 65% indicated that they never (14%), occasionally (15%), or sometimes (36%) maintain a low-fat diet. Less than two-thirds (64%) of participants indicated that they always take their antihypertensive medication exactly as prescribed by their doctors. At least 39% of participants admitted that they always or most of the time (6%) and sometimes (33%) forget to take their medications, while 27% of participants indicated that they sometimes decide not to take their blood pressure medications.

### 3.5. Bivariate Association

Table 2 reports the bivariate associations between the dependent and the independent variables. A significant negative association between adherence to medication and general beliefs about medication was detected, indicating that individuals with strong beliefs that medications are harmful and overprescribed tended to have lower medication adherence (*r* = −0.24; *p* < 0.0001). Similarly, a significant negative association was observed between the index that measured specific concern and medication adherence (*r* = −0.26; *p* < 0.0001), indicating that individuals with strong concerns about their antihypertensive medications tended to have lower medication adherence. No association between specific necessity scores and medication adherence was detected (*r* = −0.07; *p* = 0.202). Knowledge about hypertension was associated with both adherence to medication (*r* = 0.12; *p* < 0.05) and lifestyle modifications (*r* = 0.16; *p* < 0.05). Depression symptoms were associated with all scales that measured self-care and beliefs about antihypertensive medicines. However, no association between depression scale and knowledge about hypertension was detected. Finally, examination of correlations between all independent variables provides no signs of multicollinearity among the variables that were used in the multivariate liner regression models.

### 3.6. Multivariate Analysis

#### Multivariate Correlates of Adherence to Hypertension Self-Care

Multiple linear regression was carried out to investigate the relationship between adherence to antihypertensive drug regimens and adherence to recommended lifestyle modifications (Table 3). Gender was the only demographic variable that significantly correlated with both adherence to antihypertensive medication regimens and lifestyle modification. Controlling for age, education, and other relevant variables, women were more likely to adhere to both types of hypertension self-care. Older age was associated with a higher level of adherence to recommended lifestyle modifications.

Adjusting for demographic characteristics and other related variables, a higher level of adherence to antihypertensive medications was associated with (1) less financial strain, (2) a higher level of continuity of medical care, (3) less negative general beliefs about medications, (4) fewer concerns about antihypertensive medications, and (5) a higher level of hypertension knowledge. However, adjusting for demographic characteristics, (1) a higher level of knowledge about hypertension, (2) fewer depressive symptoms, and (3) better self-perceived health status were associated with a higher level of adherence to lifestyle modifications. Interestingly, while two out of three indices of belief about medicine were associated with adherence to medication regimens, none of these indices were associated with adherence to lifestyle modifications.

## 4. Discussion

This study revealed several predictive factors (beliefs, behaviors, knowledge of hypertension, and demographic factors) for adherence to both lifestyle recommendations and antihypertensive medication among African American older adults. Participants who had a higher level of knowledge regarding hypertension were more likely to adhere to both lifestyle recommendations and antihypertensive medication regimens. However, we found more predictors for adherence to medication use than lifestyle recommendations.

The rate of hypertension and its related complications and mortality are known to be more prevalent in African Americans [3,5,48,49]. Mensah et al. pointed to a 1.5 to 2 times higher rate in addition to about a 5-year reduced life expectancy in African Americans when compared to Whites with hypertension [50]. Poor adherence to antihypertensive medication and lifestyle recommendations is widely known to have adverse effects on cardiovascular-related mortality, especially in African Americans [51]. This study’s findings are consistent with current literature about the effects of these factors on lifestyle recommendations and antihypertensive medications and their eventual impact on hypertension-related complications [52,53,54].

In our study, participants who had depressive symptoms were more likely to have negative beliefs and behaviors, particularly in relation to antihypertensive medication. Depression is a usual comorbid finding in individuals with hypertension [55], with a three times greater likelihood of nonadherence to medical therapy [56]. A systematic review by Chete et al. reported eight studies that identified more than one significant negative association between depression and adherence to antihypertensive medication [57]. For example, one study conducted among 190 inner-city African American males revealed that depressive symptoms were related to poor adherence to antihypertensive medication when bivariate analysis was conducted [58]. Several studies have also concluded that symptoms of depression are significantly correlated to adherence to antihypertensive medication and that self-efficacy/confidence—the fourth construct within the Health Belief Model (HBM)—mediates this association in African Americans [59,60,61]. Though our study did not seek to determine this mediating factor, the corroborated association between depression and adherence to antihypertensive medication [56,62,63] is significant in that it allows for a wide-reaching approach to addressing both hypertension-related complications and curtailing mental health conditions such as depression in adherence to antihypertensive medication among older adults. Further interventional studies are required to address depression and adherence to antihypertensive medication and lifestyle recommendations within the older African American population.

In a study of 400 African Americans from Los Angeles, Bazargan et al. also noted that individuals with greater knowledge about the therapeutic reason for a medication regimen were close to seven times more likely to be adherent to that regimen [13]. This underscores our study’s findings of the strong association between knowledge and adherence as well as the need to educate African American older adults properly and adequately. Levine et al. advocated for utilizing a simplified, culturally sensitive approach that actively mobilizes and engages the community via community-health centered participation [64]. This approach among similar community-partnered projects has been successful in several interventions that have sought to address behavior change especially in under-resourced communities [65,66,67]. Levine and colleagues further spell out community health workers as agents of health information dissemination [50]. While this is plausible, the limited resource allocation to low income, medically underserved communities [54] makes this unlikely. There is scarce data on the cost effectiveness of running these community-health worker programs [53]. Health care providers are still considered the final, trusted source of health information [55] in these areas [54] and will, in the meantime, have to be relied upon to communicate this vital information.

Nonetheless, studies have documented short face-to-face encounters, large numbers seeking care, and provider burnout as factors that negatively affect the patient–provider interaction [68]. Providers tend not to enquire from patients about their medication-taking practices and beliefs [69]. Thus, it is imperative to empower providers to consciously engage their patients about their beliefs, knowledge, and behaviors regarding both hypertension and its treatment modalities. Providers can also provide tailored counseling to help patients improve both adherence to antihypertensive medication and lifestyle recommendations [70]. This requires education and programs that can be used to support providers. In effect, an extended and engaged contact time not only allows for misconceptions and concerns to be addressed but also fosters the patient–provider relationship [71,72]. This allows for better chances for continued care by the same provider with improved health outcomes [71,73]. Our results support the need to emphasize and increase continuity of care of African American older adults considering its significant correlation with adherence to antihypertensive medication. Enhanced adherence is only one of the many benefits of enhancing continuity of care. A recent study showed that continuity of care was protective against polypharmacy [74] and hospitalization [75]. According to The American Heart Association/American College lifestyle guidelines, lifestyle recommendations such as exercise/physical activity, dietary modification, and reductions in weight and tobacco use have been suggested as initial forms of treatment as well as an adjunct therapy for managing hypertension [76]. Excessive salt, fat, alcohol intake, and tobacco use have been implicated in the pathophysiology of cardiovascular-related conditions such as hypertension [77]. Our findings revealed that half and two-thirds of participants do not eat low-salt and low-fat diets, respectively, and that almost a third are unaware of the consequences of alcohol and tobacco use. This challenge in uptake of lifestyle recommendations for some African American older adults may be due to barriers like inadequate social support, financial constraints, lack of access to physical activity resources, and unhealthy nutritional supplies [78]. In South Central Los Angeles, where this study was carried out, a majority of its residents are low-income minorities [42] and are described as living in food deserts—areas that have limited access to grocery stores but abound in relatively unhealthy food options like fast-food restaurants and convenience stores [79,80]. The omnipresence of such unhealthy food sources impedes efforts to enhance hypertension prevention and control [48].

Our study emphasizes the need for enhanced health-related knowledge and its impact on increasing adherence to lifestyle recommendations [61,81]. According to Human Resources and Services Administration (HRSA), South Los Angeles is considered a medically underserved area that lacks the adequate number of providers required to offer healthcare education [82]. Most African Americans obtain a general understanding of hypertension and its risk factors like obesity through various forms of media including broadcast (i.e., television) and not from physicians/providers [83]. Also, the situation is made worse by an accepted cultural imagery of excess bodyweight among African Americans despite the cardiovascular complications of obesity [84]. These factors cumulatively impact the effective adoption of lifestyle recommendations among older African American adults.

This study highlights the important association between fewer depressive symptoms and higher adherence to lifestyle recommendations. Other studies have shown that African Americans are more likely than whites to exhibit depressive symptoms [85] and that depressive symptoms are related to unhealthy behaviors, particularly in individuals at risk of cardiovascular-related disease [86]. Bonnet et al. also draw attention to the many challenges of attempting to alter one’s lifestyle in the presence of depression [86].

It should be noted also that, although several public health interventions have sought to improve adherence in individuals with hypertension, those in medically underserved African American communities remain minimally tested [51].

## 5. Recommendations

It is important that an economically sustainable, culturally sensitive primary prevention approach is promoted, especially in low-income communities. It must be one that seeks to critically engage patients and providers in shared decision making via counselling if it is to be successful in increasing adherence to lifestyle recommendations. Physicians who work in underserved communities require resources like scheduled time, funding, and counseling skills to ensure effectiveness and confidence in delivering this service to their patients. A community-based approach dedicated to enlightening both providers and patients may contribute to improving the care of hypertensive patients [87,88]. Older African Americans with depression may benefit from tailored, holistic comorbid treatments that include effective follow-up and system monitoring [89].

## 6. Directions for Future Research

More research is needed on factors that impact adherence to lifestyle recommendations in addition to the assessment of counselling rates of physicians treating underserved hypertensive older African Americans. Identifying perspectives regarding adherence to lifestyle recommendations and antihypertensive medications is vital for the development and evaluation of successful intervention programs among underserved African American middle aged and older adults with hypertension.

## 7. Restrictions

The cross-sectional research design employed in this study attributes no causality between the variables analyzed. Participants were required to recollect lifestyle- and medication-related recommendations and patterns in the surveys. This could introduce recall bias. Data was obtained using a convenience sampling method; thus, results may either under- or overrepresent the African American sampling frame. Regardless of these limitations, the results obtained contributes to current literature.

## 8. Conclusions

Fewer demographic, social, behavioral, cognitive, and medical factors explain adherence to lifestyle recommendations than adherence to medication in underserved African American middle-aged and older adults with hypertension. The highly associated adherence to a medication regimen identified include less financial strain, a higher level of continuity of medical care, less negative general beliefs about medications, fewer concerns about antihypertensive medications, and a higher level of hypertension knowledge. However, a higher level of knowledge about hypertension, fewer depressive symptoms, and better self-perceived health status were associated with a higher level of adherence to lifestyle recommendations. Thus, it might be more challenging to develop a successful intervention promoting adherence to lifestyle recommendations than an intervention promoting medication adherence in this population. More research is needed on this issue

## Figures and Tables

**Table 1:** Descriptive characteristics.

Sociodemographic Factors	*n*	%
**Gender**		
Male	4-May-1900	6-Feb-1900
Female	31-Jul-1900	3-Mar-1900
**Living Alone**		
No	5-Apr-1900	28-Jan-1900
Yes	29-Aug-1900	11-Mar-1900
		**Mean ± SD**
Age (years: 55–96)		69.56 ± 9.25
Education attainment (1–16)		12.73 ± 2.13
Financial strains (1–5)		2.42 ± 1.23
Continuity of medical care (0–3)		2.32 ± 0.68
Self-rated health status (1–5)		3.28 ± 1.03
Major chronic conditions (0–9)		3.66 ± 1.99
Depression symptoms (0–14)		3.37 ± 3.04
Hypertension knowledge (2–5)		3.85 ± 0.40
Medication adherence (2.5–5)		4.34 ± 0.68
Adherence to lifestyle modification (1.4–5)		3.66 ± 0.61

**Table 2:** Bivariate correlations between all variables.

Variables	1	2	3	4	5	6	7	8	9	10	11	12	13	14
1. Age	1.00													
2. Gender	0.12 *	1.00												
3. Education	−0.19 **	0.10	1.00											
4. Living arrangement	0.17 **	0.11 *	−0.09	1.00										
5. Financial strains	−0.28 **	−0.16 *	−0.10	−0.010	1.00									
6. Continuity of care	0.19 **	0.04	0.03	0.14 *	−0.01	1.00								
7. Self-rated health	−0.18 **	−0.03	−0.09	0.04	0.30 **	−0.07	1.00							
8. Chronic conditions	0.03	0.14 *	−0.11	0.12 *	0.17 **	0.03	0.29 **	1.00						
9. Depression	−0.23 **	−0.05	−0.15 **	−0.01	0.44 **	−0.14 *	0.41 **	0.31 **	1.00					
10. General beliefs	0.00	−0.08	−0.12 *	−0.08	0.20 **	0.00	0.06	0.10	0.18 **	1.00				
11. Necessity beliefs	−0.13 *	−0.10	−0.11 *	0.06	0.14 *	−0.14 **	0.20 **	0.13 *	0.17 **	0.00	1.00			
12. Concerns beliefs	−0.08	−0.09	−0.16 **	−0.01	0.33 **	−0.03	0.12 *	0.15 **	0.23 **	0.000	0.00	1.00		
13. HBP knowledge	−0.04	−0.04	0.09	0.00	−0.07	−0.05	0.00	0.07	−0.03	0.13 *	0.01	−0.12 *	1.00	
14. Medication adherence	0.17 **	0.20 **	0.14 *	0.02	−0.34 **	0.17 **	−0.18 **	−0.14 **	−0.23 **	−0.24 **	−0.07	−0.26 **	0.12 *	1
15. Adherence to lifestyle modifications	0.29 **	0.12 *	0.05	0.07	−0.22 **	0.07	−0.31 **	−0.10	−0.37 **	−0.03	−0.09	−0.14 *	0.16 **	0.30 **

*p < 0.05; ** *p* < 0.01.

**Table 3:** Multiple linear regression between explanatory variables and blood pressure self-care scale among underserved middle-aged and older African American adults (*n* = 330).

Independent Variables	Adherence to HBP Drug Regimens			Adherence to Recommend Lifestyle Modifications		
	*B*	*Beta*	*Sig.*	*B*	*Beta*	*Sig.*
Age	0.007	0.089	0.105	0.014	0.214	0.000
Gender	0.219	0.155	0.003	0.122	0.096	0.061
Education	0.015	0.044	0.394	0.000	−0.002	0.976
Living arrangement	−0.059	−0.039	0.441	0.043	0.032	0.532
Financial strains	−0.097	−0.174	0.004	0.027	0.055	0.362
Continuity of medical cares	0.150	0.151	0.003	−0.018	−0.020	0.692
Self-rated health	−0.029	−0.043	0.436	−0.104	−0.176	0.002
Chronic conditions	−0.028	−0.082	0.132	0.004	0.015	0.790
Depressive symptoms	0.003	0.011	0.853	−0.055	−0.273	0.000
**Beliefs about Medicines**						
General Beliefs	−0.138	−0.202	0.000	−0.002	−0.004	0.945
Necessity beliefs	0.009	0.013	0.799	−0.001	−0.002	0.966
Concerns beliefs	−0.098	−0.143	0.007	−0.020	−0.033	0.540
Hypertension Knowledge	0.232	0.135	0.008	0.259	0.169	0.001
Constant	2.554		0.000	1.919		0.000
Adjusted R Square	0.224				0.222	

Sig: Significance.
